# Local stability of cooperation in a continuous model of indirect reciprocity

**DOI:** 10.1038/s41598-021-93598-7

**Published:** 2021-07-09

**Authors:** Sanghun Lee, Yohsuke Murase, Seung Ki Baek

**Affiliations:** 1 Department of Physics, Pukyong National University, Busan, 48513 Korea; 2 RIKEN Center for Computational Science, Kobe, Hyogo 650-0047 Japan

**Keywords:** Social evolution, Applied mathematics

## Abstract

Reputation is a powerful mechanism to enforce cooperation among unrelated individuals through indirect reciprocity, but it suffers from disagreement originating from private assessment, noise, and incomplete information. In this work, we investigate stability of cooperation in the donation game by regarding each player’s reputation and behaviour as continuous variables. Through perturbative calculation, we derive a condition that a social norm should satisfy to give penalties to its close variants, provided that everyone initially cooperates with a good reputation, and this result is supported by numerical simulation. A crucial factor of the condition is whether a well-reputed player’s donation to an ill-reputed co-player is appreciated by other members of the society, and the condition can be reduced to a threshold for the benefit-cost ratio of cooperation which depends on the reputational sensitivity to a donor’s behaviour as well as on the behavioural sensitivity to a recipient’s reputation. Our continuum formulation suggests how indirect reciprocity can work beyond the dichotomy between good and bad even in the presence of inhomogeneity, noise, and incomplete information.

## Introduction

Reputation was an absolutely essential asset in trade of the illiterate in the premodern era^1^, and it still plays a crucial role in markets and communities, making reputation management a central part of marketing and public relations. Also in a variety of social contexts starting from early childhood, we evaluate others based on third-party interactions^2^ and adjust our own behaviour to earn good reputations from others^3^. In this regard, although some studies suggest the existence of social evaluation in species other than humans^4^, *Homo sapiens* seems to have unique capability to use information of other social members through rumour and gossip.

Evolutionary biologists argue that the ability of social evaluation helps us extend the range of cooperation beyond kinship by encouraging cooperators and punishing defectors in a social dilemma^5–11^. A classical example of a social dilemma is the donation game, in which a player’s cooperation benefits his or her co-player by an amount of *b* at the cost of *c*, where $0<c<b$. The following payoff matrix defines the game:

1 $$\begin{aligned} \left( \begin{array}{c|cc} &{} C &{} D\\ \hline C &{} b-c &{} -c\\ D &{} b &{} 0 \end{array} \right) , \end{aligned}$$

where we abbreviate cooperation and defection as *C* and *D*, respectively. As is clearly seen from this payoff matrix, choosing *D* is the rational choice for each player whereas mutual cooperation is better for both, hence a dilemma. The players can escape from mutual defection by the action of reciprocity if the game is repeated^12–19^, but the price is that they have to remember the past and repeat interaction with sufficiently high probability, which is sometimes unfeasible. The basic idea of indirect reciprocity is that even a single encounter between two persons can be enough if that experience is reliably transferred in the form of reputation to those who will interact with these players in future. In other words, the problem is how to store, transmit, and retrieve information on each others’s past behaviour in a distributed manner^9,20^. Experiments show that the notion of indirect reciprocity provides a useful explanation for cooperative human behaviour^21,22^.

For this mechanism to work, we need two rules as a social norm: One is an assessment rule to assign reputation to a player based on his or her action to another player. The other is a behavioural rule to prescribe an action between *C* and *D*, when players’ reputations are given. An early idea was a norm called Image Scoring, which judges the donor’s *C* and *D* as good and bad, respectively^6^. According to this norm, cooperation can thrive when

2 $$\begin{aligned} q > c/b, \end{aligned}$$

where *q* means the probability of knowing someone’s reputation^23^. On the one hand, this condition seems natural because it parallels Hamilton’s rule for kin selection, and the only difference is that *q* has replaced genetic relatedness. On the other hand, if one asks what is an essential prerequisite for a norm to promote cooperation, it is not answered by Eq. (2), and we need a broader perspective on the structure of social norms.

According to Kandori’s formalism^24^, Image Scoring is an example of ‘first-order’ assessment rules because its judgment depends only on the donor’s action. A ‘second-order’ assessment rule takes the recipient’s reputation into account, and a ‘third-order’ assessment rule additionally refers to the donor’s reputation. The number of possible third-order rules thus amounts to $2^{2^3} = 256$. On the other hand, the number of actions rules is $2^{2^2} = 16$ because a behavioural rule prescribes an action depending on the reputations of the donor and recipient. Among the $2^{2^3 + 2^2} = 4096$ combinations, we have the *leading eight*^25,26^, the eight pairs of an assessment rule $\alpha$ and a behavioural rule $\beta$ that make cooperative equilibrium evolutionarily stable against every mutant with $\beta ' \ne \beta$ (Table 1).

**Table 1:** Leading eight.

Rule	$\alpha _{1C1}$	$\alpha _{1D1}$	$\alpha _{1C0}$	$\alpha _{1D0}$	$\alpha _{0C1}$	$\alpha _{0D1}$	$\alpha _{0C0}$	$\alpha _{0D0}$	$\beta _{11}$	$\beta _{10}$	$\beta _{01}$	$\beta _{00}$
L1	1	0	1	1	1	0	1	0	*C*	*D*	*C*	*C*
L2 (Consistent Standing)	1	0	0	1	1	0	1	0	*C*	*D*	*C*	*C*
L3 (Simple Standing)	1	0	1	1	1	0	1	1	*C*	*D*	*C*	*D*
L4	1	0	1	1	1	0	0	1	*C*	*D*	*C*	*D*
L5	1	0	0	1	1	0	1	1	*C*	*D*	*C*	*D*
L6 (Stern Judging)	1	0	0	1	1	0	0	1	*C*	*D*	*C*	*D*
L7 (Staying)	1	0	1	1	1	0	0	0	*C*	*D*	*C*	*D*
L8 (Judging)	1	0	0	1	1	0	0	0	*C*	*D*	*C*	*D*

Cooperation and defection are denoted as *C* and *D*, respectively, and a player’s reputation is either good (1) or bad (0). By $\alpha _{uXv}$, we mean the reputation assigned to a player who did $X \in \{C, D\}$ with reputation *u* to another player with reputation *v*. The behavioural rule $\beta _{uv}$ prescribes what a player should do between *C* and *D* when he or she has reputation *u* and the co-player has reputation *v*. We note that L1 has been known as Contrite Tit-for-Tat in the context of direct reciprocity^27–30^.

The situation becomes complicated when reputations are not globally shared in the population: Misjudgement does occur in the presence of error, and some players may even have their own private rules of assessment^31–34^. Then, strict social norms such as ‘Judging’ and ‘Stern Judging’ completely fail to tell if other players are good or bad, although they successfully induce cooperation when reputation is always public information^35,36^. Communication rounds can be introduced to resolve disagreements^10^, or one may need empathy or prudence in judgment to alleviate the problem^37,38^, but these remedies imply the intrinsic instability of the reputation mechanism in its pure sense. We also point out that most of the existing models are based on an assumption that the dynamic variables are binary, although reputation is not really a simple dichotomy between good and bad, and some actions cannot be classified as either cooperation or defection^39,40^.

In this work, we thus wish to investigate indirect reciprocity by taking reputations and actions as continuous variables. By doing so, we can naturally deal with the continuous dynamics between the existing norm and its close variants by means of analytic tools. We also expect that this formulation can be used to address the problems of error and incompleteness: The idea is that perception error will effectively replace a binary reputation by a probabilistic mixture between good and bad, just as a binary action can be replaced by a probabilistic mixture of cooperation and defection in the presence of implementation error. Although the number of possible social norms expands to infinity, we will restrict ourselves to local-stability analysis by assuming that mutants appear from a small neighbourhood of the existing social norm.

## Analysis

Let us imagine a large population and denote the number of players as *N*. The basic setting is that a random pair of players are picked up to play the donation game [Eq. (1)]. In our model, the player chosen as a donor decides the degree of cooperation to the co-player between zero and one, which mean full defection and full cooperation, respectively, based on their reputations. Let $m_{ij}$ denote player *j*’s reputation from the viewpoint of player *i*. The player *i* also has a behavioural rule $\beta _i (m_{ii}, m_{ij})$, which determines how much he or she will do as a donor to *j*. Note that all of $m_{ij}$, $\alpha _{i}$, and $\beta _i$ for any *i* and *j* take real values inside the unit interval. Player *k* is observing the interaction between *i* and *j*, and it has its own assessment rule $\alpha _k (m_{ki}, \beta _i, m_{kj})$. With observation probability $q > 0$, the reputation that *k* assigns to *i* will be updated on average as follows:

3 $$\begin{aligned} m_{ki}^{t+1} = (1-q) m_{ki}^t + \frac{q}{N-1} \sum _{j \ne i} \alpha _k \left[ m_{ki}^t, \beta _i \left( m_{ii}^t, m_{ij}^t \right) , m_{kj}^t \right] \end{aligned}$$

where the superscripts have been used as time indices. Equation (3) is to be analysed in this section. Before proceeding, let us note two points: First, as a deterministic equation, Eq. (3) does not include error explicitly. If the probability of error is low, Eq. (3) will nevertheless describe the dynamics for most of the time, and the main effect of error will be to perturb the output of $\alpha$ or $\beta$ by a small amount at a point in time, say, $t=0$. Second, from a mathematical point of view, it is preferable to treat both diagonal and off-diagonal elements on an equal footing as in Eq. (3), which implies that one has to observe even the self-reputation $m_{ii}$ probabilistically. If that sounds unrealistic, we may alternatively assume that donors and recipients update their self-reputations with probability one. However, it is a reasonable guess that the difference between these two settings becomes marginal when *N* is large enough, and this guess is indeed verified by numerical calculation (not shown).

Throughout this work, $\alpha$ and $\beta$ are assumed to be C$^2$-differentiable. In addition, we will focus on the cases where the system has a fixed point characterized by

4a $$\begin{aligned} \alpha (1,1,1)&=1 \end{aligned}$$

4b $$\begin{aligned} \beta (1,1)&=1 \end{aligned}$$

because otherwise the norm would not sustain cooperation among well-reputed players from the start. As concrete examples of $\alpha$ and $\beta$, let us extend the leading eight to deal with continuous variables by applying the trilinear (bilinear) interpolation to $\alpha$ ($\beta$) in Table 1. If we consider L3 (Simple Standing), for instance, it is described by

5a $$\begin{aligned} \alpha _\text {SS}(x,y,z)&= yz - z + 1 \end{aligned}$$

5b $$\begin{aligned} \beta _\text {SS}(x,y)&= y. \end{aligned}$$

If we define $A_\xi \equiv \left. \partial \alpha / \partial \xi \right| _{(1,1,1)}$ and $B_\lambda \equiv \left. \partial \beta / \partial \lambda \right| _{(1,1)}$ with $\xi \in \{x,y,z\}$ and $\lambda \in \{x,y\}$, all the leading eight have $A_y = B_y = 1$, together with $A_x = B_x = 0$, and these are related with the basic properties of the leading eight to be nice, retaliatory, apologetic, and forgiving^26^.

Below, we will examine two aspects of stability: The first is recovery of full cooperation from disagreement in a homogeneous population where everyone uses the same $\alpha$ and $\beta$^36^. Starting from $m_{ij}=1$ for every *i* and *j*, the dynamics of Eq. (3) will be investigated within the framework of linear-stability analysis. The second aspect is the stability against mutant norms, for which we have to check the long-term payoff difference between the resident and mutant norms in a stationary state. We again start this analysis from a nearly homogeneous population in which only one individual considers using a slightly different norm. Although private assignment of reputation is allowed, the point is that it will remain unrealised if no one has a reason to deviate from the prevailing norm, considering that such deviation will only decrease his or her own payoff. In this sense, the homogeneity serves as a self-consistent assumption in the second part of the stability analysis.

### Recovery from disagreement

To understand the time evolution of disagreement in a homogeneous population with common $\alpha$ and $\beta$, let us rewrite Eq. (3):

6 $$\begin{aligned} m_{ki}^{t+1} = (1-q) m_{ki}^t + \frac{q}{N-1} \sum _{j \ne i} \alpha \left[ m_{ki}^t, \beta \left( m_{ii}^t, m_{ij}^t \right) , m_{kj}^t \right] , \end{aligned}$$

where $\alpha _k = \alpha$ and and $\beta _i = \beta$ in this homogeneous population. Initially, everyone starts with a good reputation, which can be perturbed by error. To see whether the magnitude of the perturbation grows with time, we set $m_{ki}^t \equiv 1-\varepsilon _{ki}^t$ and expand the above equation to the first order of $\varepsilon$ as follows:

7 $$\begin{aligned} 1-\varepsilon _{ki}^{t+1}= & {} (1-q) \left( 1-\varepsilon _{ki}^t \right) + \frac{q}{N-1} \sum _{j \ne i} \alpha \left[ 1-\varepsilon _{ki}^t, \beta \left( 1-\varepsilon _{ii}^t, 1-\varepsilon _{ij}^t \right) , 1-\varepsilon _{kj}^t \right] \end{aligned}$$

8 $$\begin{aligned}\approx & {} (1-q) \left( 1-\varepsilon _{ki}^t \right) + \frac{q}{N-1} \sum _{j \ne i} \alpha \left[ 1-\varepsilon _{ki}^t, 1- \left( B_x\varepsilon _{ii}^t + B_y \varepsilon _{ij}^t \right) , 1-\varepsilon _{kj}^t \right] \end{aligned}$$

9 $$\begin{aligned}\approx & {} (1-q) \left( 1-\varepsilon _{ki}^t \right) + \frac{q}{N-1} \sum _{j \ne i} \left\{ 1- \left[ A_x \varepsilon _{ki}^t + A_y \left( B_x\varepsilon _{ii}^t + B_y \varepsilon _{ij}^t \right) + A_z \varepsilon _{kj}^t \right] \right\} , \end{aligned}$$

or, equivalently,

10 $$\begin{aligned} \varepsilon _{ki}^{t+1}\approx & {} (1-q) \varepsilon _{ki}^t + \frac{q}{N-1} \sum _{j \ne i} [{A_x} \varepsilon _{ki}^t + {A_y} ({B_x} \varepsilon _{ii}^t + {B_y} \varepsilon _{ij}^t) + {A_z} \varepsilon _{kj}^t] \end{aligned}$$

11 $$\begin{aligned}= & {} (1-q + q{A_x}) \varepsilon _{ki}^t + q {A_y} {B_x} \varepsilon _{ii}^t + \frac{q}{N-1} \sum _{j \ne i} [{A_y} {B_y} \varepsilon _{ij}^t + {A_z} \varepsilon _{kj}^t], \end{aligned}$$

which leads to

12 $$\begin{aligned} \frac{d}{dt} \varepsilon _{ki} \approx -q(1-{A_x}) \varepsilon _{ki} + q {A_y} {B_x} \varepsilon _{ii} + \frac{q}{N-1} \sum _{j \ne i} [{A_y} {B_y} \varepsilon _{ij} + {A_z} \varepsilon _{kj}], \end{aligned}$$

if time is regarded as a continuous variable. This is a linear-algebraic system with an $N^2 \times N^2$ matrix. In principle, we can find the stability at the origin as well as the speed of convergence toward it by calculating the eigenvalues. By attempting this calculation from $N=2$ to 5 with a symbolic-algebra system^41^, we see the following pattern in the eigenvalue structure:

13 $$\begin{aligned} \Lambda _1^{(N^2-2N+1)}= & {} q\left( -1+{A_x}- \frac{1}{N-1}{A_z} \right) \end{aligned}$$

14 $$\begin{aligned} \Lambda _2^{(N-1)}= & {} q(-1+{A_x}+{A_z}) \end{aligned}$$

15 $$\begin{aligned} \Lambda _3^{(N-1)}= & {} q\left( -1+{A_x}-\frac{1}{N-1}{A_z}+{A_y} {B_x} -\frac{1}{N-1}{A_y} {B_y} \right) \end{aligned}$$

16 $$\begin{aligned} \Lambda _4^{(1)}= & {} q(-1 + {A_x} + {A_z} + {A_y} {B_x} + {A_y} {B_y}), \end{aligned}$$

where each superscript on the left-hand side means multiplicity of the corresponding eigenvalue. Based on this observation, we conjecture that this pattern is valid for general *N*. A sufficient condition for recovery to take place in this first-order calculation is that the largest eigenvalue is negative. The largest eigenvalue is the last one, $\Lambda _4^{(1)}$, because all the derivatives are non-negative. In other words, the first-order perturbation analysis gives a sufficient condition for local recovery as

17 $$\begin{aligned} Q \equiv -1 + {A_x} + {A_z} + {A_y} ({B_x} + {B_y}) < 0. \end{aligned}$$

### Suppression of mutants

To analyse the effect of a mutant norm, we will look at the long-time behaviour in Eq. (3). That is, for given sets of rules $\{ \alpha _i \}$ and $\{ \beta _i \}$, we assume that the image matrix $\{ m_{ij} \}$ will converge to a stationary state as $t \rightarrow \infty$, satisfying

18 $$\begin{aligned} m_{ki} = \frac{1}{N-1} \sum _{j \ne i} \alpha _k \left[ m_{ki}, \beta _i (m_{ii}, m_{ij}), m_{kj} \right] . \end{aligned}$$

Note that *q* only affects the speed of convergence to stationarity: It is an irrelevant parameter as far as we work with a stationary state, which is in contrast with Eq. (2), where *q* appears as an essential condition for indirect reciprocity. In the donation game with benefit *b* and cost *c* [Eq. (1)], player *j*’s expected payoff can be computed as

19 $$\begin{aligned} \pi _j = \frac{1}{N-1} \left[ b \sum _{i \ne j} \beta _i (m_{ii}, m_{ij}) - c \sum _{i \ne j} \beta _j (m_{jj}, m_{ji}) \right] . \end{aligned}$$

For the sake of simplicity, let us assume that every person with index 1 to $N-1$ has the same rules and equal reputation, so that player $i=1$ is representative for all of them in the resident population. Now, the situation is effectively reduced to a two-body problem between players 0 and 1. By assumption, the system initially starts from a fully cooperative state where everyone has good reputation, i.e., $m_{11} = \beta (1,1) = \alpha (1,1,1)= 1$. The rules used by the resident population will be denoted by $\alpha \equiv \alpha _1$ and $\beta \equiv \beta _1$ without the subscripts. Now, the focal player 0 attempts a slightly different norm, defined by $\alpha _0 (x,y,z) = \alpha (x,y,z) - \delta (x,y,z)$ and $\beta _0(x,y) = \beta (x,y) - \eta (x,y)$ with $|\delta | \ll 1$ and $|\eta | \ll 1$. Let us assume that the introduction of $\delta$ and $\eta$ causes small changes in the image matrix: Only the elements related to the focal player will be affected because the residents can still give $m_{11}=1$ to each other when the mutant occupies a negligible fraction of the population, i.e., $N \gg 1$. Therefore, if mutation leads to $m_{00} = 1-\varepsilon _{00}$, $m_{01} = 1-\varepsilon _{01}$, and $m_{10} = 1-\varepsilon _{10}$ with $\varepsilon _{ij} \ll 1$, by expanding Eq. (18) to the linear order of perturbation (see Methods), we obtain

20 $$\begin{aligned} \varepsilon _{00}= & {} \frac{(1-{A_x}+{A_y} {B_y})\delta _1 + (1-{A_x}-{A_z}){A_y} \eta _1}{(1-{A_x}-{A_z})(1-{A_x}-{A_y} {B_x})} \end{aligned}$$

21 $$\begin{aligned} \varepsilon _{01}= & {} \frac{\delta _1}{1-{A_x}-{A_z}} \end{aligned}$$

22 $$\begin{aligned} \varepsilon _{10}= & {} \frac{({B_x}+{B_y})\delta _1 + (1-{A_x}-{A_z})\eta _1}{(1-{A_x}-{A_z})(1-{A_x}-{A_y} {B_x})} {A_y}, \end{aligned}$$

where $\delta _1 \equiv \delta (1,1,1) \ge 0$ and $\eta _1 \equiv \eta (1,1) \ge 0$, provided that

23 $$\begin{aligned} {A_x} + {A_z}< & {} 1 \end{aligned}$$

24 $$\begin{aligned} {A_x} + {A_y} {B_x}< & {} 1. \end{aligned}$$

We can now calculate the focal player 0’s payoff as follows:

25 $$\begin{aligned} \pi _0= & {} \frac{1}{N-1} \left[ b \sum _{i \ne 0} \beta _i (m_{ii}, m_{i0}) - c \sum _{i \ne 0} \beta _0 (m_{00}, m_{0i}) \right] \end{aligned}$$

26 $$\begin{aligned}= & {} b \beta (m_{11}, m_{10}) - c\beta _0 (m_{00}, m_{01}) \end{aligned}$$

27 $$\begin{aligned}\approx & {} b \left( 1- {B_y} \varepsilon _{10} \right) - c \left( 1-{B_x} \varepsilon _{00} - {B_y} \varepsilon _{01} - \eta _1 \right) . \end{aligned}$$

If we plug Eqs. (20), (21), and (22) here, the payoff change $\Delta \pi _0 \equiv \pi _0 - (b-c)$ is given as

28 $$\begin{aligned} \Delta \pi _0 = -\frac{b{A_y} {B_y} - c(1-{A_x})}{1-{A_x} -{A_y} {B_x}} \left[ \left( \frac{{B_x}+{B_y}}{1-{A_x}-{A_z}} \right) \delta _1 + \eta _1 \right] , \end{aligned}$$

and we require this quantity to be negative for any small positive $\delta _1$ and $\eta _1$. Here, it is worth stressing that the signs of $\delta _1$ and $\eta _1$ are determined because we start from a fully cooperative state with $m_{ij}=1$: For other states where $\delta$ and $\eta$ can take either sign, the first-order terms should vanish so that the second-order terms can determine the sign of $\Delta \pi _0$. In this respect, the payoff analysis is greatly simplified by choosing the specific initial state. Because of Eqs. (23) and (24), the negativity of Eq. (28) is reduced to the following inequality:

29 $$\begin{aligned} \frac{b}{c} > \frac{1-{A_x}}{{A_y} {B_y}}, \end{aligned}$$

which, together with Eqs. (23) and (24), characterizes a condition for a social norm to stabilize cooperation against local mutants, as an alternative to Eq. (2). This result is intuitively plausible because cooperation will be unstable if one does not lose reputation by decreasing the degree of cooperation (i.e., ${A_y} \approx 0$) or if no punishment is imposed on an ill-reputed player (i.e., ${B_y} \approx 0$).

Two remarks are in order: First, whether mutation occurs to a single individual or to a fraction of the population does not alter the final result in this first-order calculation. Suppose that the population is divided into two groups with fractions *p* and $1-p$, respectively. One group has $\alpha$ and $\beta$, and the other group has $\alpha +\delta$ and $\beta +\eta$. Then, the payoff difference between two players, each from a different group, is still the same as Eq. (28) (see Methods). Therefore, if an advantageous mutation occurs with $p \ll 1$, the mutants are always better off than the resident until they take over the whole population, i.e., $p \rightarrow 1$. In this sense, our condition determines not only the initial invasion but also the fixation of a mutant norm, as long as it is a close variant of the resident one. Second, one could ask what happens if a mutant differs only in the slopes while keeping $\delta _1=\eta _1=0$. Equation (28) does not answer this question because it is based on an assumption that the $\left. \partial \delta / \partial \xi \right| _{(1,1,1)} \varepsilon _{ij}$ and $\left. \partial \eta / \partial \lambda \right| _{(1,1)} \varepsilon _{ij}$, where $\xi \in \{ x, y, z\}$ and $\lambda \in \{x, y\}$, are all negligibly small in the first-order calculation. However, even if the derivatives are taken into consideration, we find that $\delta _1$ or $\eta _1$ must still be positive to make a finite payoff change. In other words, the basic form of Eq. (28) is still useful, although the coefficients include correction terms. The performance of such a ‘slope mutant’ will be checked numerically at the end of the next section.

## Results

In this section, we will numerically check the continuous-reputation system in the presence of inhomogeneity, noise, and incomplete information. More specifically, the simulation code should allow each player *i* to adopt a different set of $\alpha _i$ and $\beta _i$ to simulate an inhomogeneous population. The outputs of $\alpha _i$ and $\beta _i$ can be affected by random-number generation to simulate a noisy environment where misperception and misimplementation occur, and every interaction between a pair of players will update only some part of the reputation system, parametrized by the observation probability *q*, because information is incomplete.

Our numerical simulation code is based on a publicly available one^36^ but has been modified to handle continuous variables. To simulate the dynamics of a society of *N* players, we work with an $N \times N$ image matrix $\{ m_{ij} \}$ whose elements are all set to be ones at the beginning. Every player starts with zero payoff, i.e., $\pi _i = 0$ initially. In each round, we randomly pick up two players, say, *i* and *j*, so that *i* is the donor and *j* is the recipient of the donation game [Eq. (1)], which has $b=2$ and $c=1$ unless otherwise noted. Each other member of the population, say, *k*, independently observes the interaction with probability *q* and updates $m_{ki}$ according to his or her own assessment rule $\alpha _k$. Although the above analyses are generally applicable to any norms defined by $\alpha$ and $\beta$ as long as Eq. (4) is true, we would like to focus on Simple Standing as a representative example of successful norms. Misperception may occur with probability *e*, whereby $m_{ki}$ becomes a random number drawn from the unit interval. Implementation error is also simulated in a similar way by setting the output of $\beta$ to a random number between zero (defection) and one (cooperation) with probability $\gamma$. This process is repeated for *M* rounds, during which every player’s payoff is accumulated. Equation (18) suggests that *q* will only affect the convergence rate toward a stationary state. For this reason, we will fix this parameter at $q=0.4$ throughout the simulation unless otherwise mentioned. Note also that we have deliberately made this parameter low enough to violate the inequality in Eq. (2).

**Figure 1:**
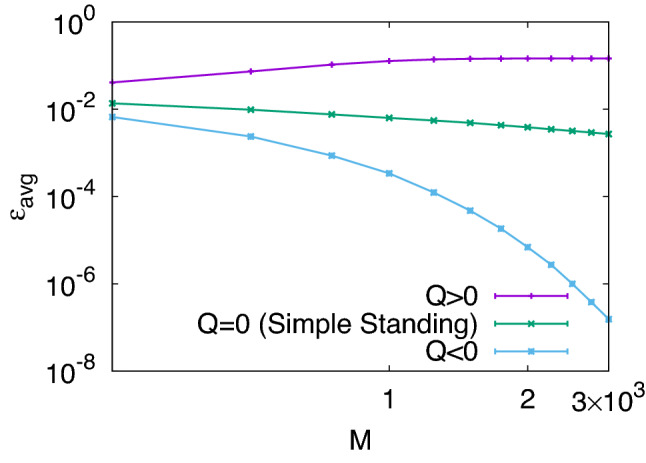
Recovery from disagreement when *M* rounds have elapsed in a population of size $N=50$ with common $\alpha$ and $\beta$. Initially, we randomly pick up $20\%$ of the image-matrix elements and change them to 0.9, whereas the rest of them remain as 1’s, and the simulation has been repeated over $10^3$ independent samples without error, i.e., $e=\gamma =0$. In this log-log plot, the vertical axis shows the average difference from the state of perfect reputation, represented by the average of $\varepsilon _{ki} \equiv 1-m_{ki}$. We have tested three norms, which all have $\alpha (1,1,1)=1$ and $\beta (1,1)=1$ but differ in their local slopes there: The first norm has $({A_x}, {A_y}, {A_z}) = (0.2,0.9,0.1)$ and $({B_x}, {B_y}) = (0.2,0.8)$, which together yield $Q \equiv -1 + {A_x} + {A_z} + {A_y} ({B_x} + {B_y})>0$ [Eq. (17)]. The next one is Simple Standing with $({A_x}, {A_y}, {A_z}) = (1,0,1)$ and $({B_x}, {B_y}) = (0,1)$, which has $Q=0$. The last one for $Q<0$ is a variant of Simple Standing with $({A_x}, {A_y}, {A_z}) = (0,0.9,0)$ and $({B_x}, {B_y}) = (0,0.9)$.

To see the effect of *Q* on recovery [Eq. (17)], we have tested three norms one by one in a homogeneous population with $e=\gamma =0$ (Fig. 1). All these norms have $\alpha (1,1,1)=1$ and $\beta (1,1)=1$ in common but their local slopes are different to make *Q* positive, zero, or negative. The first norm under consideration has $({A_x}, {A_y}, {A_z}) = (0.2,0.9,0.1)$ and $({B_x}, {B_y}) = (0.2,0.8)$, which together make $Q>0$. If some members of the population initially have slightly imperfect reputations, they fail to recover under such a norm. If $Q<0$, on the other hand, the recovery process indeed takes place with a finite time scale. Although Simple Standing violates Eq. (17) by having $Q=0$, our simulation shows that it gets reputation recovered with the aid of higher-order terms, and it is a slow process with a diverging time scale. Among the leading eight, L1, L3 (Simple Standing), L4, and L7 (Staying) fall into this category of $Q=0$, whereas the other four, i.e., L2 (Consistent Standing), L5, L6 (Stern Judging), and L8 (Judging), have positive *Q*. The difference between these two groups is whether ${A_z} = \alpha _{1C1} - \alpha _{1C0} = 1-\alpha _{1C0}$ is zero or one: If a well-reputed player has to risk his or her own reputation in helping an ill-reputed co-player, i.e., $\alpha _{1C0}=0$, it means ${A_z}=1$ and $Q>0$, so we can conclude that the initial state of $m_{ki} \approx 1$ will not be recovered. According to an earlier study on the leading eight^36^, the latter four with $Q>0$ have long recovery time from a single disagreement in reputation. Although it is not derived from a continuum formulation, the result is qualitatively consistent with ours.

**Figure 2:**
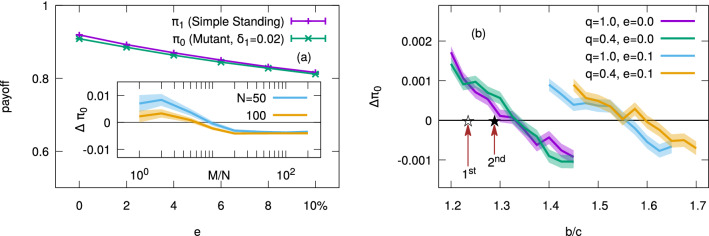
Stationary states of a population with $$N=50$$ players, reached from an initial condition with $$m_{ij}=1$$ for every *i* and *j*. In each case, the mutant norm differs from the resident one by $$\delta _1 = 0.02$$ and occupies one half of the population ($$p=0.5$$). The game is defined by Eq. (1) with $$b=2$$ and $$c=1$$. (**a**) Average payoffs over $$5\times$$$$10^4$$ samples when the resident norm is Simple Standing. Everyone can observe each interaction with probability $$q=0.4$$, and perception error and implementation error occur with probabilities $$e = 0.1$$ and $$\gamma = 0.1$$, respectively. Inset: Convergence of payoff difference $$\Delta \pi _0 \equiv \pi _0 - \pi _1$$ as *M* increases. If $$M \propto gN$$ with a sufficiently large constant $$g \gtrsim O(10)$$, the mutants will obtain less payoffs than Simple Standing, making $$\Delta \pi _0 < 0$$. This result has no significant dependence on *N*. (**b**) Payoff advantage of mutants with respect to the resident as a function of *b*/*c*, averaged over $$5\times 10^4$$ samples per each, when $$M=10^4$$. The resident norm, a variant of Simple Standing, has $$\alpha (1,1,1)=\beta (1,1)=1$$ and $${A_x} = {A_z} = {B_x} = 0$$ but $${A_y} = {B_y} = 0.9$$ as in Fig. 1. Implementation error occurs with probability $$\gamma =0.1$$, and the results are qualitatively the same for any small $$\gamma$$. The stars on the horizontal line indicate the predicted threshold values obtained from the first-order and second-order calculations, respectively. In both of these panels, the shaded areas represent error bars.

As for the effect of mutation in assessment rules, let us consider the following scenario: One half of the population have adopted Simple Standing [Eq. (5)], whereas the other half are “mutants” using a different assessment rule $\alpha _\text {SS} - \delta$ with

30 $$\begin{aligned} \delta (x,y,z) = \delta _1 (2yz - 2z + 1), \end{aligned}$$

where $\delta _1$ is a small number, say, $\delta _1 = 0.02$ in numerical calculation. Such a half-and-half configuration is being used because the payoff difference [Eq. (28)] is unaffected by the fraction of mutants, *p* (see Methods). Figure 2a shows that the level of cooperation is still high if $e \ll 1$, and the cooperation rate of Simple Standing in the continuous form converges to $100\%$ in a monomorphic population (not shown). Furthermore, we see that mutants are worse off than the players of Simple Standing, i.e., $\pi _0 < \pi _1$, as expected.

From a theoretical viewpoint, an important question is how quickly the mutants’ payoff difference $\Delta \pi _0 \equiv \pi _0 - \pi _1$ becomes negative: Although we have argued that the inequality will be true for Simple Standing, the calculation is based on several assumptions. In particular, one could say that Eq. (3) corresponds to $M \propto N^2$ because it seems to assume that everyone meets every other player with a weighting factor of $1/(N-1)$. If $M \propto N^2$, however, it would pose a serious obstacle to applying such a norm to the society where the number of interactions will grow linearly with *N*. Fortunately, the inset of Fig. 2a shows that $M \propto N$ indeed suffices to make $\Delta \pi _0$ negative. One could also point out that the payoff difference should be $\Delta \pi _0 = - \delta _1$ according to Eq. (28), whereas the result in Fig. 2a has smaller magnitude. A part of the reason is that Eq. (28) does not take perception error into account, so the numerical value recovers the predicted order of magnitude as $e \rightarrow 0$. In addition, Eq. (28) is based on a first-order approximation, and a higher-order calculation reproduces the observed value with greater precision (see Methods).

An important prediction of our analysis is the threshold of *b*/*c* to make a local mutant worse off than the resident population [Eq. (29)]. In Fig. 2b, we directly check Eq. (29) by measuring payoffs in equilibrium in a population of size $N=50$. A variant of Simple Standing is chosen as the resident norm, which occupies $p=0.5$ of the population with $\alpha (1,1,1)=\beta (1,1)=1$ and ${A_x} = {A_z} = {B_x} = 0$. The only difference from Simple Standing is that ${A_y} = {B_y} = 0.9$, and the reason of this variation is that the first-order perturbation for the leading eight develops spurious singularity when *p* is finite (see Methods). When perception is free from error, i.e., $e=0$, the results do not depend on the observation probability *q*, as expected from stationarity [Eq. (18)], and the threshold value is consistent with the first- and second-order calculations [the arrows in Fig. 2b]. When $e>0$, on the other hand, the threshold is pushed upward, implying that cooperation becomes harder to stabilize because of the perception error. In addition, we now see that incomplete information with $q<1$ can shift the threshold further with the aid of positive *e*. We have also changed the value of $\gamma$, but it does not not change the average behaviour in the above results. Overall, the point of Fig. 2b is that our analysis does capture the correct picture.

**Figure 3:**
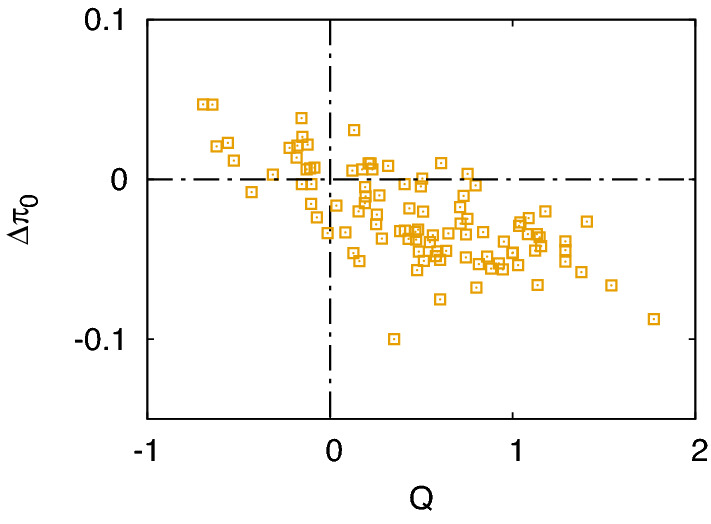
Payoff difference between the resident population using Simple Standing and its ‘slope mutant’, which has the same $\beta$ and $\alpha (1,1,1)=1$ but different slopes ${A_x}$ and ${A_y}$ and ${A_z}$. Each point denotes a randomly generated mutant through the trilinear interpolation among $\alpha (1,1,1)=1$ and seven random values $\alpha (0,0,0), \alpha (0,0,1), \ldots , \alpha (1,1,0)$ within the unit interval. The mutant norm occupies $10\%$ of the whole population whose size is $N=100$. The horizontal axis shows the mutant’s *Q*-value [Eq. (17)], and the vertical axis means its payoff difference $\Delta \pi _0$ with respect to the resident norm after a sufficiently long time, e.g., $M/N \sim O(10^3)$. As before, the game is defined with $b=2$ and $c=1$, and the observation probability is $q=0.4$. Perception error and implementation error occur with probabilities $e=0.1$ and $\gamma =0.1$, respectively.

Finally, we can numerically check the effect of a ‘slope mutant’, which has $\alpha (1,1,1)=1$ as a fixed point and the same behavioural rule as Simple Standing but differs in the slopes ${A_x}$, ${A_y}$ and ${A_z}$. To be more specific, let us assume that a mutant norm occupies $10\%$ of the population whereas the rest of them are using Simple Standing. The values of $\alpha (x,y,z)$ at the vertices of the three-dimensional unit hypercube are randomly drawn from the unit interval, except for $\alpha (1,1,1)=1$. Then, the trilinear interpolation is used to construct the continuous assessment rule. According to our simulation (Fig. 3), the performance of the mutant norm is strongly correlated with its *Q*-value [Eq. (17)]. Recall that the expression of *Q* has been derived in the context of recovery from small disagreement in a homogeneous population. Figure 3 nevertheless suggests that it can also serve as a useful indicator to tell if a minority of ‘slope mutants’ will be competitive with the resident norm, even when the difference between their assessment rules is not necessarily small.

## Summary and discussion

In summary, we have studied indirect reciprocity with private, noisy, and incomplete information by extending the binary variables for reputation and behaviour to continuous ones. The extension to continuum is an idealization because it would impose an excessive cognitive burden to keep track of others' reputations without discretization; nonetheless, this abstraction allows us to overcome the fact that the sharp dichotomy between good and bad is often found insufficient in reporting an assessment^42–44^. In particular, this formulation makes it possible to check the role of sensitivity to new information in judging others and adjusting our own behaviour. That is, according to Eq. (29), the benefit-cost ratio of cooperation should increase for stabilizing the cooperative initial state, if reputation is insensitive to observed behaviour (low ${A_y}$) or if the level of cooperation is insensitive to the recipient’s reputation (low ${B_y}$). At the same time, in contrast to the well-known condition for indirect reciprocity akin to Hamilton’s rule [Eq. (2)], we have observed that incompleteness of information, controlled by $q<1$, mainly affects the convergence toward a stationary state without altering the overall conclusion. This approach sheds light on difference among the leading eight in their recovery speeds from a single disagreement. Our analysis has identified the key factor $\alpha _{1C0}$ in Table 1, i.e., how to assign reputation to a well-reputed donor who chooses *C* against an ill-reputed recipient: If this choice is regarded as good according to $\alpha _{1C0}=1$, making the assessment function $\alpha (x,y,z)$ insensitive to *z*, the recovery can take place smoothly. As a result, we conclude that $\alpha$ should respond to the donor’s defection (${A_y}>0$) but not necessarily to the players’ reputations (e.g., ${A_x} = {A_z} = 0$). A recent study also argues that helping an ill-reputed player should be regarded as good to maintain stable cooperation^45^. Such understanding of indirect reciprocity in terms of sensitivity is important because, as is usual, information processing through reputation has a trade-off between robustness and sensitivity: One could underestimate new information and fail to adapt, or, one could overestimate it and fail to distinguish noise from the signal. In practice, the best way of assessment seems to be updating little by little upon arrival of new information^46^, and such a possibility is already incorporated in this continuum formulation.

It should be emphasized that our analysis has focused on local perturbation to the existing norm. Therefore, our inequalities cannot be interpreted as a condition for evolutionary stability against every possible mutant. Moreover, although $\Delta \pi _0$ is found independent of *p* in our analysis, one should keep in mind that it results from a first-order theory so that higher-order corrections generally show dependence on *p*. If a mutant is sufficiently different from the resident, then the first-order theory fails and the payoff difference may well depend on *p*. For instance, if we think of a population consisting of L1 and L8 (Table 1), we see that L1 is better off only when it comprises the majority of the population (not shown). Having said that, our local analysis can nevertheless provide a necessary condition which will hold for stronger notions of stability as well. We also believe that this locality assumption is usually plausible in reality, considering that a social norm is a complex construct that combines expectation and action in a mutually reinforcing manner and thus resists change but small ones^47^. An empirical analysis shows that even orthographic and lexical norms change so slowly that it takes centuries unless intervened by a formal institution^48^. Another restriction in our analytic approach is that the mutation is assumed to have positive $\delta _1$ so that the mutant is not fully content with the initial cooperative state. If two norms have $\delta _1=0$ in common and differ only by slopes at the initial state, the first-order perturbation does not give a definite answer as to their dynamics. Having positive $\delta _1$ can be interpreted from a myopic player’s point of view as follows: A selfish player in a cooperating population may feel tempted to devalue others’ cooperation and reduce his or her own cost of cooperation toward them. If our condition is met, however, such behaviour will eventually be punished by the social norm.

“Maturity of mind is the capacity to endure uncertainty,” says a maxim. Although one lesson of the life is that we have to accept the grey area between good and bad, reputation is still something that can be easily driven to extremes, and what is worse is that it often goes in a different direction for each observer. Despite the theoretical achievement of indirect reciprocity, its real difficulties are thus manifested in the problem of private assessment, noise, and incomplete information. Our finding suggests that we can get a better grip on indirect reciprocity by preparing reputational and behavioural scales with finer gradations, which may be thought of as a form of systematic deliberation to protect each other’s reputation from rash judgement.

## Methods

### Linear-order corrections

Equation (18) in the large-*N* limit is written as follows:

31 $$\begin{aligned} m_{00}= & {} \frac{1}{N-1} \sum _{j\ne 0} \alpha _0 [m_{00}, \beta _0 (m_{00}, m_{0j}), m_{0j}] = \alpha _0 [m_{00}, \beta _0 (m_{00}, m_{01}), m_{01}] \end{aligned}$$

32 $$\begin{aligned} m_{01}= & {} \frac{1}{N-1} \sum _{j\ne 1} \alpha _0 [m_{01}, \beta _1 (m_{11}, m_{1j}), m_{0j}] \approx \alpha _0 [m_{01}, \beta _1 (m_{11}, m_{11}), m_{01}] \end{aligned}$$

33 $$\begin{aligned} m_{10}= & {} \frac{1}{N-1} \sum _{j\ne 0} \alpha _1 [m_{10}, \beta _0 (m_{00}, m_{0j}), m_{1j}] = \alpha _1 [m_{10}, \beta _0 (m_{00}, m_{01}), m_{11}] \end{aligned}$$

34 $$\begin{aligned} m_{11}= & {} \frac{1}{N-1} \sum _{j\ne 1} \alpha _1 [m_{11}, \beta _1 (m_{11}, m_{1j}), m_{1j}] \approx \alpha _1 [m_{11}, \beta _1 (m_{11}, m_{11}), m_{11}]. \end{aligned}$$

With $m_{00} = 1-\varepsilon _{00}$, $m_{01} = 1-\varepsilon _{01}$, and $m_{10} = 1-\varepsilon _{10}$, Eq. (32) becomes

35 $$\begin{aligned} 1-\varepsilon _{01}= & {} \alpha _0 (1-\varepsilon _{01}, 1, 1-\varepsilon _{01}) = \alpha (1-\varepsilon _{01}, 1, 1-\varepsilon _{01}) - \delta (1-\varepsilon _{01}, 1, 1-\varepsilon _{01}) \end{aligned}$$

36 $$\begin{aligned}\approx & {} \alpha (1,1,1) - {A_x} \varepsilon _{01} - {A_z} \varepsilon _{01} - \delta (1,1,1) = 1 - {A_x} \varepsilon _{01} - {A_z} \varepsilon _{01} - \delta _1, \end{aligned}$$

where $\alpha _\xi \equiv \left. \partial \alpha / \partial \xi \right| _{(1,1,1)}$ and $\delta _1 \equiv \delta (1,1,1)$. Thus, we have

37 $$\begin{aligned} \varepsilon _{01} \approx \left( 1-{A_x} - {A_z} \right) ^{-1} \delta _1. \end{aligned}$$

Likewise,

38 $$\begin{aligned} \beta _0 (1-\varepsilon _{00}, 1-\varepsilon _{01})= & {} \beta (1-\varepsilon _{00}, 1-\varepsilon _{01}) - \eta (1-\varepsilon _{00}, 1-\varepsilon _{01}) \end{aligned}$$

39 $$\begin{aligned}\approx & {} 1 - {B_x} \varepsilon _{00} - {B_y} \varepsilon _{01} - \eta _1, \end{aligned}$$

where $\beta _\lambda \equiv \left. \partial \beta / \partial \lambda \right| _{(1,1)}$ and $\eta _1 \equiv \eta (1,1)$. Using this expression, we obtain from Eq. (33) the following:

40 $$\begin{aligned} 1-\varepsilon _{10}= & {} \alpha \left( 1-\varepsilon _{10}, 1-{B_x} \varepsilon _{00} - {B_y} \varepsilon _{01} -\eta _1, 1 \right) \end{aligned}$$

41 $$\begin{aligned}\approx & {} 1- {A_x} \varepsilon _{10} - {A_y} \left( {B_x} \varepsilon _{00} + {B_y} \varepsilon _{01} + \eta _1 \right) , \end{aligned}$$

which means

42 $$\begin{aligned} \varepsilon _{10}= & {} \frac{{A_y}}{1-{A_x}} ({B_x} \varepsilon _{00} + {B_y} \varepsilon _{01} + \eta _1 ) \end{aligned}$$

43 $$\begin{aligned}= & {} \frac{{A_y}}{1-{A_x}} \left[ {B_x} \varepsilon _{00} + {B_y} (1-{A_x} - {A_z})^{-1} \delta _1 + \eta _1 \right] . \end{aligned}$$

To get a closed-form expression for this, we need $\varepsilon _{00}$ in addition to $\varepsilon _{01}$ [Eq. (37)]. Thus, from Eq. (31), we derive

44 $$\begin{aligned} 1-\varepsilon _{00}\approx & {} \alpha \left[ 1-\varepsilon _{00}, \beta _0 (1-\varepsilon _{00}, 1-\varepsilon _{01}), 1-\varepsilon _{01} \right] - \delta _1 \end{aligned}$$

45 $$\begin{aligned}\approx & {} \alpha \left( 1-\varepsilon _{00}, 1-{B_x} \varepsilon _{00} - {B_y} \varepsilon _{01} - \eta _1, 1-\varepsilon _{01} \right) - \delta _1 \end{aligned}$$

46 $$\begin{aligned}\approx & {} 1 - {A_x} \varepsilon _{00} - {A_y} \left( {B_x} \varepsilon _{00} + {B_y} \varepsilon _{01} + \eta _1 \right) - {A_z} \varepsilon _{01} - \delta _1, \end{aligned}$$

which gives

47 $$\begin{aligned} \varepsilon _{00}= & {} \frac{1}{1-{A_x} - {A_y} {B_x}} \left[ ({A_y} {B_y} + {A_z}) \varepsilon _{01} + {A_y} \eta _1 + \delta _1 \right] \end{aligned}$$

48 $$\begin{aligned}= & {} \frac{1}{1-{A_x} - {A_y} {B_x}} \left[ \frac{{A_y} {B_y} + {A_z}}{1-{A_x} - {A_z}} \delta _1 + {A_y} \eta _1 + \delta _1 \right] , \end{aligned}$$

where we have used Eq. (37). By substituting Eq. (48) into Eq. (43), we can write $\varepsilon _{10}$ explicitly.

### Finite fraction of mutants

If a mutant norm occupies a finite fraction *p*, Eqs. (31) to (34) are generalized to

49 $$\begin{aligned} m_{00}= & {} p \alpha _0 [m_{00}, \beta _0(m_{00}, m_{00}), m_{00}] + {\bar{p}} \alpha _0 [m_{00}, \beta _0(m_{00}, m_{01}), m_{01}] \end{aligned}$$

50 $$\begin{aligned} m_{01}= & {} p \alpha _0 [m_{01}, \beta _1(m_{11}, m_{10}), m_{00}] + {\bar{p}} \alpha _0 [m_{01}, \beta _1(m_{11}, m_{11}), m_{01}] \end{aligned}$$

51 $$\begin{aligned} m_{10}= & {} p \alpha _1 [m_{10}, \beta _0(m_{00}, m_{00}), m_{10}] + {\bar{p}} \alpha _1 [m_{10}, \beta _0(m_{00}, m_{01}), m_{11}] \end{aligned}$$

52 $$\begin{aligned} m_{11}= & {} p \alpha _1 [m_{11}, \beta _1(m_{11}, m_{10}), m_{10}] + {\bar{p}} \alpha _1 [m_{11}, \beta _1(m_{11}, m_{11}), m_{11}], \end{aligned}$$

where ${\bar{p}} \equiv 1-p$. Through linearisation, the above equations are rewritten as

53 $$\begin{aligned} 1-\varepsilon _{00}\approx & {} p [1-{A_x} \varepsilon _{00} - {A_y} ({B_x} \varepsilon _{00} + {B_y} \varepsilon _{00} + \eta _1) - {A_z} \varepsilon _{00} - \delta _1]\nonumber \\&+ {\bar{p}} [1-{A_x} \varepsilon _{00} - {A_y} ({B_x} \varepsilon _{00} + {B_y} \varepsilon _{01} + \eta _1) - {A_z} \varepsilon _{01} - \delta _1] \end{aligned}$$

54 $$\begin{aligned} 1-\varepsilon _{01}\approx & {} p [1-{A_x} \varepsilon _{01} - {A_y} ({B_x} \varepsilon _{11} + {B_y} \varepsilon _{10}) - {A_z} \varepsilon _{00} - \delta _1]\nonumber \\&+ {\bar{p}} [1-{A_x} \varepsilon _{01} - {A_y} ({B_x} \varepsilon _{11} + {B_y} \varepsilon _{11}) - {A_z} \varepsilon _{01} - \delta _1] \end{aligned}$$

55 $$\begin{aligned} 1-\varepsilon _{10}\approx & {} p [1-{A_x} \varepsilon _{10} - {A_y} ({B_x} \varepsilon _{00} + {B_y} \varepsilon _{00} + \eta _1) - {A_z} \varepsilon _{10}]\nonumber \\&+ {\bar{p}} [1-{A_x} \varepsilon _{10} - {A_y} ({B_x} \varepsilon _{00} + {B_y} \varepsilon _{01} + \eta _1) - {A_z} \varepsilon _{11}] \end{aligned}$$

56 $$\begin{aligned} 1-\varepsilon _{11}\approx & {} p [1-{A_x} \varepsilon _{11} - {A_y} ({B_x} \varepsilon _{11} + {B_y} \varepsilon _{10}) - {A_z} \varepsilon _{10}]\nonumber \\&+ {\bar{p}} [1-{A_x} \varepsilon _{11} - {A_y} ({B_x} \varepsilon _{11} + {B_y} \varepsilon _{11}) - {A_z} \varepsilon _{11}]. \end{aligned}$$

After some algebra, we find

57 $$\begin{aligned} \varepsilon _{00}= & {} \frac{\delta _1 \left\{ {A_x}^2+{A_x} ({A_y} {B_x}+{A_z}-2)-{\bar{p}}{A_y}^2 {B_x} {B_y}-{\bar{p}}{A_y}^2 {B_y}^2+{A_z} [{A_y} (p{B_x}-{\bar{p}}{B_y})-1]-{A_y} {B_x}+1\right\} }{(1-{A_x}-{A_z}) (1-{A_x}-{A_y} {B_x}) (1-{A_x}-{A_y} {B_x}-{A_y} {B_y}-{A_z})}\nonumber \\&+\frac{{A_y} \eta _1 (1-{A_x}-{A_z}) (1-{A_x}-{A_y} {B_x} -{\bar{p}}{A_y} {B_y}-{\bar{p}}{A_z})}{(1-{A_x}-{A_z}) (1-{A_x}-{A_y} {B_x}) (1-{A_x}-{A_y} {B_x}-{A_y} {B_y}-{A_z})} \end{aligned}$$

58 $$\begin{aligned} \varepsilon _{01}= & {} \frac{{A_y} \eta _1 p (1-{A_x}-{A_z}) ({A_y} {B_y}+{A_z})}{(1-{A_x}-{A_z}) (1-{A_x}-{A_y} {B_x}) (1-{A_x}-{A_y} {B_x}-{A_y} {B_y}-{A_z})}\nonumber \\&+\frac{\delta _1 \left[ {A_x}^2+{A_x} (2 {A_y} {B_x}+{A_y} {B_y}+{A_z}-2)+{A_y}^2 {B_x}^2+{A_y}^2 {B_x} {B_y} p+{A_y}^2 {B_x} {B_y}+{A_y}^2 {B_y}^2 p \right] }{(1-{A_x}-{A_z}) (1-{A_x}-{A_y} {B_x}) (1-{A_x}-{A_y} {B_x}-{A_y} {B_y}-{A_z})}\nonumber \\&+\frac{\delta _1 \left\{ {A_z} [{A_y} (p{B_x}+{B_x}+p{B_y})-1]-2 {A_y} {B_x}-{A_y} {B_y}+1\right\} }{(1-{A_x}-{A_z}) (1-{A_x}-{A_y} {B_x}) (1-{A_x}-{A_y} {B_x}-{A_y} {B_y}-{A_z})} \end{aligned}$$

59 $$\begin{aligned} \varepsilon _{10}= & {} \frac{{A_y} (1-{A_x}-{A_y} {B_x}-{\bar{p}}{A_y} {B_y}-{\bar{p}}{A_z}) [\eta _1 (1-{A_x}-{A_z})+({B_x} +{B_y}) \delta _1]}{(1-{A_x}-{A_z}) (1-{A_x}-{A_y} {B_x}) (1-{A_x}-{A_y} {B_x}-{A_y} {B_y}-{A_z})} \end{aligned}$$

60 $$\begin{aligned} \varepsilon _{11}= & {} \frac{{A_y} p ({A_y} {B_y}+{A_z}) (\eta _1 (1-{A_x}-{A_z})+({B_x} +{B_y}) \delta _1)}{(1-{A_x}-{A_z}) (1-{A_x}-{A_y} {B_x}) (1-{A_x}-{A_y} {B_x}-{A_y} {B_y}-{A_z})}, \end{aligned}$$

from which one can reproduce the previous results [Eqs. (20) to (22)] by taking the limit of $p \rightarrow 0$. The denominators seem to require another inequality in addition to Eqs. (23) and (24), that is,

61 $$\begin{aligned} {A_x} + {A_z} + {A_y} ({B_x} + {B_y}) < 1, \end{aligned}$$

which is equivalent to Eq. (17). Recall that the continuous versions of the leading eight always have ${A_y} = {B_y} = 1$ and ${A_x} = {B_x} = 0$ in common, which means that they all violate this inequality. However, in practice, no singularity arises for Simple Standing if higher-order corrections are included, and even the second-order calculation agrees moderately well with numerical results.

The payoff earned by a mutant is calculated as

62 $$\begin{aligned} \pi _0= & {} b [p \beta _0 (m_{00}, m_{00}) + (1-p) \beta _1(m_{11}, m_{10})] \nonumber \\&-c [p \beta _0 (m_{00}, m_{00}) + (1-p) \beta _0(m_{00}, m_{01})] \end{aligned}$$

63 $$\begin{aligned}\approx & {} b [p (1-{B_x} \varepsilon _{00} - {B_y} \varepsilon _{00} - \eta _1) + (1-p)(1-{B_x} \varepsilon _{11} - {B_y} \varepsilon _{10})]\nonumber \\&-c [p (1-{B_x} \varepsilon _{00} - {B_y} \varepsilon _{00} - \eta _1) + (1-p) [1-{B_x} \varepsilon _{00} - {B_y} \varepsilon _{01} - \eta _1]], \end{aligned}$$

whereas a resident player earns

64 $$\begin{aligned} \pi _1= & {} b [p \beta _0 (m_{00}, m_{01}) + (1-p) \beta _1(m_{11}, m_{11})] \nonumber \\&-c [p \beta _1 (m_{11}, m_{10}) + (1-p) \beta _1(m_{11}, m_{11})] \end{aligned}$$

65 $$\begin{aligned}\approx & {} b [p (1-{B_x} \varepsilon _{00} - {B_y} \varepsilon _{01} - \eta _1) + (1-p)(1-{B_x} \varepsilon _{11} - {B_y} \varepsilon _{11})]\nonumber \\&-c [p(1-{B_x} \varepsilon _{11} - {B_y} \varepsilon _{10}) + (1-p) (1-{B_x} \varepsilon _{11} - {B_y} \varepsilon _{11})]. \end{aligned}$$

If we plug Eqs. (57) to (60) here, the payoff difference $\Delta \pi _0 = \pi _0 - \pi _1$ becomes identical to Eq. (28) with no dependence on *p*.

### Second-order corrections

We assume that $\delta$, $\eta$, as well as their partial derivatives, and $\varepsilon _{ij}$’s are small parameters of the same order of magnitude. The second-order perturbation for $\beta _1$ can thus be written as follows:

66 $$\begin{aligned} \beta _1 (m_{11}, m_{1j})= & {} \beta (1-\varepsilon _{11}, 1-\varepsilon _{1j}) \end{aligned}$$

67 $$\begin{aligned}\approx & {} 1 - {B_x} \varepsilon _{11} - {B_y} \varepsilon _{1j} + \frac{1}{2} {B_{xx}} \varepsilon _{11}^2 + {B_{xy}} \varepsilon _{11} \varepsilon _{1j} + \frac{1}{2} {B_{yy}} \varepsilon _{1j}^2 \end{aligned}$$

68 $$\begin{aligned}\equiv & {} 1 - \kappa _1. \end{aligned}$$

Here, we write $\kappa _1 \equiv \kappa _1^{(1)} + \kappa _1^{(2)}$, where $\kappa _1^{(1)} \equiv {B_x} \varepsilon _{11} + {B_y} \varepsilon _{1j}$ and $\kappa _1^{(2)} \equiv - \left( \frac{1}{2} {B_{xx}} \varepsilon _{11}^2 + {B_{xy}} \varepsilon _{11} \varepsilon _{1j} + \frac{1}{2} {B_{yy}} \varepsilon _{1j}^2 \right)$ are first- and second-order corrections, respectively, and $B_{\mu \nu } \equiv \left. \partial ^2 \beta /\partial \mu \partial \nu \right| _{(1,1)}$. Likewise,

69 $$\begin{aligned} \beta _0 (m_{00}, m_{0j})= & {} \beta (m_{00}, m_{0j}) - \eta (m_{00}, m_{0j}) \end{aligned}$$

70 $$\begin{aligned}= & {} \beta (1-\varepsilon _{00}, 1-\varepsilon _{0j}) - \eta (1-\varepsilon _{00}, 1-\varepsilon _{0j}) \end{aligned}$$

71 $$\begin{aligned}\approx & {} \left( 1 - {B_x} \varepsilon _{00} - {B_y} \varepsilon _{0j} + \frac{1}{2} {B_{xx}} \varepsilon _{00}^2 + {B_{xy}} \varepsilon _{00} \varepsilon _{0j} + \frac{1}{2} {B_{yy}} \varepsilon _{0j}^2 \right) \nonumber \\&- \left( \eta _1 - \eta _x \varepsilon _{00} - \eta _y \varepsilon _{0j} \right) \end{aligned}$$

72 $$\begin{aligned}\equiv & {} 1 - \kappa _0, \end{aligned}$$

where $\kappa _0 \equiv \kappa _0^{(1)} + \kappa _0^{(2)}$ with $\kappa _0^{(1)} \equiv {B_x} \varepsilon _{00} + {B_y} \varepsilon _{0j} + \eta _1$ and $\kappa _0^{(2)} \equiv -\left( \frac{1}{2} {B_{xx}} \varepsilon _{00}^2 + {B_{xy}} \varepsilon _{00} \varepsilon _{0j} + \frac{1}{2} {B_{yy}} \varepsilon _{0j}^2 \right) - (\eta _x \varepsilon _{00} + \eta _y \varepsilon _{0j})$.

The second-order perturbation for $\alpha _1$ is also straightforward:

73 $$\begin{aligned} \alpha _1 [m_{1i}, \beta _i (m_{ii}, m_{ij}), m_{1j}]\approx & {} \alpha (1-\varepsilon _{1i}, 1-\kappa _i, 1-\varepsilon _{1j}) \end{aligned}$$

74 $$\begin{aligned}\approx & {} 1 - {A_x} \varepsilon _{1i} - {A_y} \kappa _i - {A_z} \varepsilon _{1j} + \frac{1}{2} {A_{xx}} \varepsilon _{1i}^2 + \frac{1}{2} {A_{yy}} \left( \kappa _i^{(1)} \right) ^2 + \frac{1}{2} {A_{zz}} \varepsilon _{1j}^2\nonumber \\&+ {A_{xy}} \varepsilon _{1i} \kappa _i^{(1)} + {A_{yz}} \kappa _i^{(1)} \varepsilon _{1j} + {A_{zx}} \varepsilon _{1i} \varepsilon _{1j}, \end{aligned}$$

where $A_{\mu \nu } \equiv \left. \partial ^2 \alpha / \partial \mu \partial \nu \right| _{(1,1,1)}$, and similarly,

75 $$\begin{aligned} \alpha _0 [m_{0i}, \beta _i (m_{ii}, m_{ij}), m_{0j}]\approx & {} \alpha (1-\varepsilon _{0i}, 1-\kappa _i, 1-\varepsilon _{0j}) - \delta (1-\varepsilon _{0i}, 1-\kappa _i, 1-\varepsilon _{0j}) \end{aligned}$$

76 $$\begin{aligned}\approx & {} \left[ 1 - {A_x} \varepsilon _{0i} - {A_y} \kappa _i - {A_z} \varepsilon _{0j} + \frac{1}{2} {A_{xx}} \varepsilon _{0i}^2 + \frac{1}{2} {A_{yy}} \left( \kappa _i^{(1)} \right) ^2 + \frac{1}{2} {A_{zz}} \varepsilon _{0j}^2 \right. \nonumber \\&\left. + {A_{xy}} \varepsilon _{0i} \kappa _i^{(1)} + {A_{yz}} \kappa _i^{(1)} \varepsilon _{0j} + {A_{zx}} \varepsilon _{0i} \varepsilon _{0j} \right] \nonumber \\&- \left( \delta _1 - \delta _x \varepsilon _{0i} - \delta _y \kappa _i^{(1)} - \delta _z \varepsilon _{0j} \right) . \end{aligned}$$

## Data Availability

The source code for this study is available at https://github.com/yohm/sim_game_continuous_reputation.
